# Tumor induced-osteomalacia caused by a phosphaturic mesenchymal tumor in the sartorius muscle: a case report

**DOI:** 10.3389/fendo.2026.1814853

**Published:** 2026-04-17

**Authors:** Weronika Rymon Lipinska, Natalia Filckowska, Marcin Ekman, Jarosław Kobiela

**Affiliations:** 1 Faculty of Medicine, Medical University of Gdansk, Gdansk, Poland; 2 Department of Surgical Oncology, Transplant Surgery and General Surgery, Faculty of Medicine, Medical University of Gdansk, Gdansk, Poland

**Keywords:** hypophosphatemia, phosphaturic mesenchymal tumor, soft tissue tumor, tumor resection, tumor-induced osteomalacia

## Abstract

Tumor-induced osteomalacia (TIO) is a rare paraneoplastic syndrome resulting from excessive secretion of fibroblast growth factor-23 (FGF23) by phosphaturic mesenchymal tumors (PMTs). We report the case of a 35-year-old man with a three-year history of progressive musculoskeletal symptoms and severe mobility impairment, who had not received a correct diagnosis during this period. Laboratory evaluation revealed severe hypophosphatemia and elevated FGF23 levels. Magnetic resonance imaging (MRI) and ^68^Ga-DOTA-TATE PET/CT identified a metabolically active lesion in the sartorius muscle. Surgical resection of the tumor resulted in complete resolution of symptoms, restoration of mobility and normalization of phosphate metabolism. This case emphasizes the importance of considering TIO in patients with unexplained osteomalacia and highlights the diagnostic value of functional imaging.

## 1 Introduction

Phosphaturic mesenchymal tumors (PMTs) have been reported in fewer than 500 cases worldwide and are often associated with significant diagnostic delay due to nonspecific clinical presentation (1). They are the most common cause of TIO, which is characterized by renal phosphate wasting, hypophosphatemia and defective bone mineralization (2).

The pathophysiology of TIO involves excessive secretion of FGF23, which decreases renal phosphate reabsorption and suppresses 1α-hydroxylase activity, leading to reduced synthesis of 1,25-dihydroxyvitamin D (3).

The clinical presentation is typically subtle and non-specific, including progressive skeletal pain, muscle weakness and recurrent insufficiency fractures, which may culminate in severe functional impairment, including inability to ambulate independently (2, 4). These features contribute to a prolonged diagnostic delay and high rate of misdiagnosis, reported in up to 95% of cases (1).

PMTs can develop in any anatomical location, but most commonly arise in bones, paranasal sinuses, feet, and thighs (5). Accurate localization is challenging due to their small size and clinically silent growth, but functional imaging targeting somatostatin receptor expression plays a crucial role in tumor detection (6). Intramuscular localization is rarely specified and appears to be uncommon in the published literature. The present case demonstrates a PMT arising within skeletal muscle, illustrating the broad anatomical variability of these tumors.

## 2 Case presentation

A 35-year-old Polish man presented with a three-year history of progressive bone pain and proximal muscle weakness. Over time, he developed impaired balance, leading to multiple low-impact falls and several pathologic fractures, including the right femoral neck, left femur, and ribs detected on PET imaging. His functional impairment worsened, leading to severe mobility limitations. Gradually, he became increasingly withdrawn from social and professional activities.

Approximately 19 months before the definitive diagnosis, the patient underwent multiple evaluations and hospitalizations in rheumatology, neurology and nephrology units due to persistent musculoskeletal complaints. Despite extensive work-up, clinicians were unable to identify the underlying cause. During this period, he was found to have severe osteoporosis, with a femoral neck T-score of –4.4 as assessed by densitometry and was even referred to a genetic counseling clinic. His symptoms persisted and progressively worsened, eventually requiring crutches for ambulation. Ongoing laboratory abnormalities, including persistent hypophosphatemia, led to referral to a tertiary center, where careful clinical assessment and broad diagnostic thinking resulted in a strong suspicion of TIO, prompting targeted evaluation. The overall diagnostic course and sequence of clinical events are summarized in **Table 1**.

**Table 1:** Timeline of the diagnostic process and clinical course.

Time from symptom onset	Clinical events and diagnostic milestones
0 months	Onset of progressive musculoskeletal pain and proximal muscle weakness
12 months	First laboratory abnormalities detected (hypophosphatemia, elevated alkaline phosphatase)
19 months	Multiple specialist evaluations (rheumatology, neurology, nephrology); no definitive diagnosis established
24–30 months	Progressive functional decline with low-impact fractures and increasing mobility impairment
36 months	Referral to tertiary center; clinical suspicion of tumor-induced osteomalacia (TIO)
36 months	Targeted diagnostic work-up initiated, including biochemical assessment and imaging
36 months	Whole-body ^68^Ga-DOTA-TATE PET/CT performed; localization of metabolically active lesion
36 months	Surgical resection of the tumor (R0 resection)
Postoperative period	Rapid normalization of serum phosphate and FGF23 levels; significant clinical improvement
Follow-up	Sustained biochemical remission and restoration of independent mobility

The physical examination revealed a firm, non-tender, immobile soft-tissue mass in the upper right thigh, with intact overlying skin and no inflammatory changes. Muscle strength was reduced, and gait was impaired. No regional lymphadenopathy was noted.

Laboratory evaluation (**Table 2**) showed hypophosphatemia (serum phosphate 1.6 mg/dL), low-normal calcium (8.5 mg/dL), elevated alkaline phosphatase (447 U/L), normal parathyroid hormone (24 pg/mL) and normal level of creatinine (0.81 mg/dL). FGF23 was markedly elevated (3916 pg/mL). Vitamin D analysis revealed normal 25-hydroxyvitamin D (34.5 ng/mL) and decreased 1,25-dihydroxyvitamin D (15.3 pg/mL), consistent with renal phosphate wasting and compatible with TIO.

**Table 2:** Laboratory test results before and after tumor resection.

Laboratory parameter	Normal Range	Pre-operation	Post-operation
Serum phosphate	2.5–4.5 mg/dL	1.6 ↓	5.2 ↑
Calcium	9.1–10.5 mg/dL	8.5 ↓	9.6
Parathyroid hormone	11–67 pg/mL	24	57.4
Alkaline phosphatase	50–116 U/L	447 ↑	338 ↑
Fibroblast Growth Factor 23	23.2–95.4 pg/mL	3916 ↑	45.49
25-hydroxyvitamin D	30–50 ng/mL	34.5	23.5 ↓
Creatinine	0.73–1.18 mg/dL	0.81	0.74

↑: above normal levels; ↓: below normal levels.

**Figure 1:**
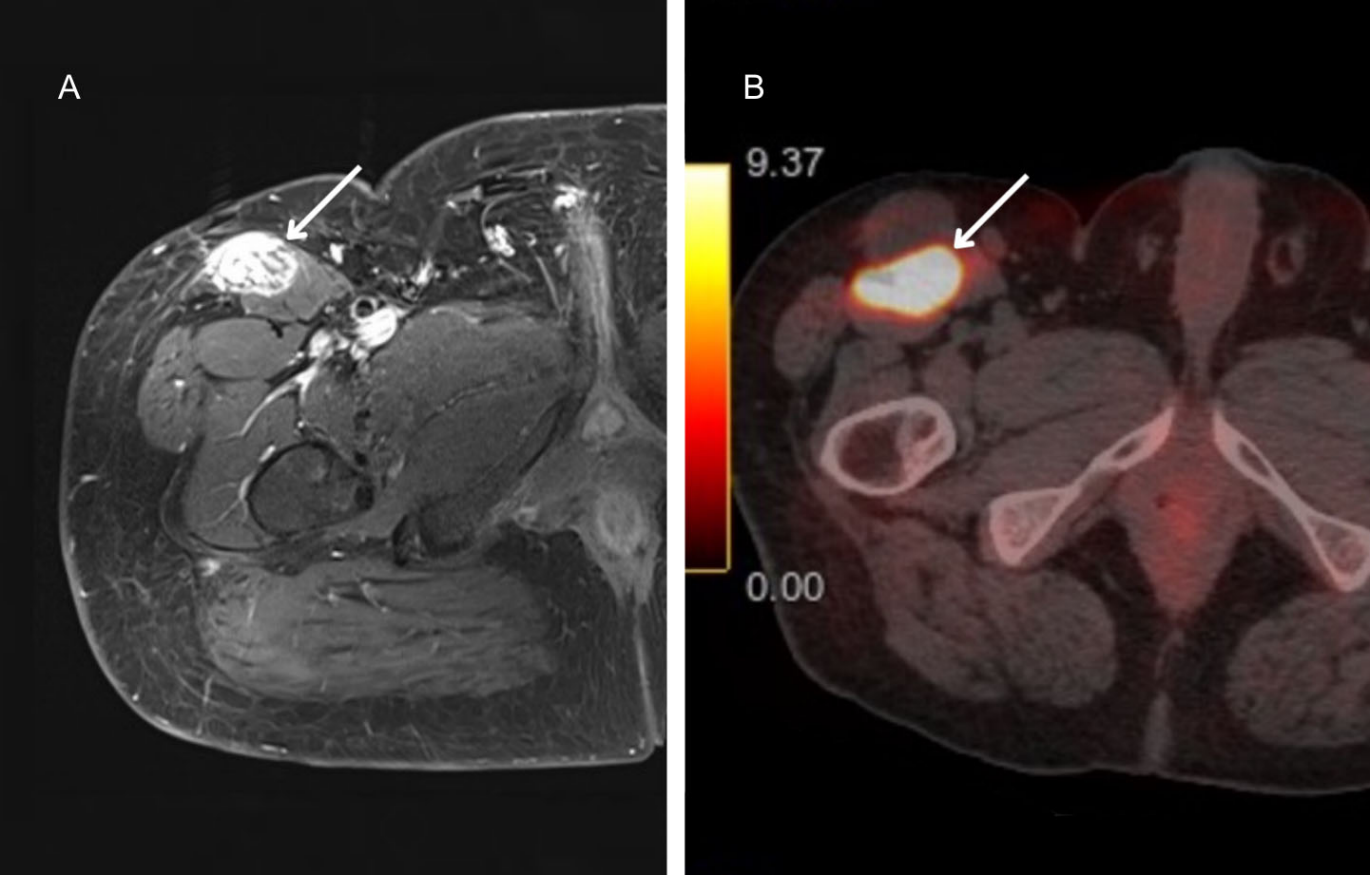
Imaging evaluation of the tumor: **(A)** Axial magnetic resonance imaging of the right thigh demonstrating a heterogeneous, well-circumscribed mass measuring 47 × 40 × 24 mm in the proximal anterolateral portion of the sartorius muscle with intense, heterogeneous post-contrast enhancement (white arrow). **(B)**^68^Ga-DOTA-TATE PET/CT demonstrates intense radiotracer uptake (42 × 41 mm; SUVmax 33.2) within the proximal right thigh lesion, with no evidence of distant metastatic disease (white arrow).

MRI of the right thigh (**Figure 1A**) demonstrated a well-circumscribed, heterogeneous soft-tissue lesion located adjacent to and partially involving the proximal anterolateral sartorius muscle, measuring 47 × 40 × 24 mm. After contrast administration, the lesion exhibited intense but heterogeneous enhancement without diffusion restriction. No additional soft-tissue or bone lesions were observed. Pelvic lymph nodes were normal in size and morphology.

The core needle biopsy revealed a mesenchymal tumor composed of branching capillary vessels and bland spindle-shaped cells arranged in a pericytoma-like pattern with foci of basophilic matrix and no necrosis. Immunohistochemistry showed CD31 (focal +), CD10 (–), SMA (±), SOX10 (–), STAT6 (–), ALK (–), desmin (-), CD34 (–), Ki-67 5%. These findings suggested a benign or low-grade mesenchymal neoplasm, with PMT remaining in the differential diagnosis.

Considering the clinical and biochemical presentation, a whole body ^68^Ga-DOTA-TATE PET/CT (**Figure 1B**) was performed to localize the phosphaturic tumor and confirm the absence of additional lesions. The scan demonstrated a metabolically somatostatin receptor-avid lesion measuring 42 × 41 mm in the upper right thigh near the inguinal region, with SUVmax 33.2, showing intense somatostatin receptor expression consistent with a somatostatin receptor-positive PMT.

Based on biochemical and imaging findings, the patient was qualified for surgical excision. Through a longitudinal proximal anteromedial thigh incision, a well-vascularized, solid mass within the upper sartorius muscle was resected en bloc with a cuff of macroscopically healthy tissue, following oncologic principles for low-grade mesenchymal tumors. The lesion was closely related to but did not infiltrate major neurovascular structures. Meticulous hemostasis and layered closure were achieved. The postoperative course was uneventful, and the patient was mobilized on the first postoperative day.

Histopathology confirmed complete (R0) excision with negative margins (0.5 cm inferiorly, 1.4 cm right, 1.6 cm left). Microscopy revealed a highly vascular lesion with staghorn-type vessels and scattered osteoclast-like giant cells. Immunohistochemistry (CD56+, ERG+, SATB2+, CK AE1/AE3–, S100–, CD34–, SOX10–) correlated with biopsy results, confirming the diagnosis of phosphaturic mesenchymal tumor.

Follow-up laboratory assessment demonstrated normalization of phosphate metabolism, with serum phosphate increasing from 1.6 mg/dL preoperatively to 5.2 mg/dL and FGF23 decreasing to 45.49 pg/mL. Creatinine remained within normal limits throughout follow-up (0.74 mg/dL). The patient reported marked improvement in quality of life, complete resolution of pain, and returned to independent ambulation within weeks after surgery.

## 3 Patient’s perspective

The patient reported a substantial improvement in quality-of-life following surgery, with complete resolution of pain and restoration of independent mobility. He no longer requires a wheelchair and is able to leave his home independently. The patient also reported a noticeable increase in muscle strength and overall physical function. He noted that the diagnostic process was prolonged and associated with progressive functional limitations and multiple inconclusive evaluations, and expressed satisfaction with the final diagnosis and treatment outcome.

## 4 Discussion

PMTs secrete phosphatonins, particularly FGF23, which reduce phosphate reabsorption in the proximal tubule of the kidney and inhibit the production of 1,25-dihydroxycholecalciferol by 1*α*-hydroxylase (7). Overexpression of phosphatonins leads to increased phosphate excretion by the kidneys, increased bone resorption with impaired mineralization, reduced osteoblast mineralization, and decreased absorption of calcium and phosphate from the gastrointestinal tract. The combined effect of these pathological mechanisms results in symptomatic osteomalacia (8).

Laboratory investigations reveal a characteristic pattern in patients with PMT, including hypophosphatemia, hyperphosphaturia, normocalcemia or calcium levels at the lower end of the normal range, elevated alkaline phosphatase levels with normal or low vitamin D3 levels, normal parathyroid hormone levels, preserved renal function, and elevated FGF23 levels (1).

The primary diagnostic investigation in PMT is imaging studies. Conventional radiological examinations often reveal indistinct margins and may resemble benign bone lesions or osteomalacic changes. Furthermore, PMTs can occur at various anatomical locations, complicating their detection. While ^18^F-FDG PET/CT is commonly used for tumor localization, it frequently fails to identify PMTs due to their small size and low metabolic activity. Therefore, ^68^Ga-DOTA-TATE PET/CT or ^68^Ga-DOTATOC PET/CT is preferred for comprehensive whole-body assessment and accurate tumor localization (9, 10). Computed tomography (CT) with contrast enhancement, used in preliminary diagnosis, often highlights fat or calcification within the tumor. MRI depicts PMT as isointense on T1-weighted sequences and hyperintense on T2-weighted sequences (11).

Tumor identification can be challenging and time-consuming, as the small size and atypical location of the lesion often result in a significant delay of several years between the onset of initial symptoms and the establishment of a diagnosis (12).

The varying clinical presentation necessitates a histopathological diagnosis of the tumor. Histopathological features of PMT typically include vessels resembling hemangiopericytoma, minimal mitotic activity, and cellular diversity, including spindle cells and giant cells. Granular calcifications and microcystic changes are often observed. Immunohistochemically, PMTs may express FGF23 and somatostatin receptor 2A, although these characteristics are not specific to this entity (13). In the differential diagnosis of PMT, solitary fibrous tumors, mesenchymal chondrosarcoma, and paraganglioma are considered. Solitary fibrous tumors lack the characteristic matrix and calcifications, while chondrosarcomas exhibit malignant round cells, and paragangliomas have a distinctive nested architecture and neuroendocrine markers that are absent in PMT. Rare cases of histologically malignant PMTs can occur, displaying sarcomatous features and suggesting potential progression from benign to malignant forms (14).

From an etiopathological perspective, the only fully effective treatment method is surgery. As a causal treatment, it eliminates the source of the humoral factors responsible for the disease’s clinical symptoms. Tumor resection completely alleviates symptoms resulting from FGF23’s biochemical and physical effects (15). FGF23 serum levels can serve as a diagnostic tool for monitoring tumor recurrence (16). Bone remineralization can occur shortly after tumor resection. In rare cases, for non-operable or difficult- to-localize tumors, alternative or adjuvant methods are recommended. Traditional treatment involves administering neutral phosphates and active vitamin D supplements. For patients not eligible for surgical treatment, burosumab, a fully human monoclonal antibody against FGF23, is used. This drug blocks FGF23 activity, allowing the kidneys to reabsorb phosphate and restore normal serum phosphate levels (17). Although such replacement therapy does not cure the disease, it provides biochemical and symptomatic improvement (15). Cryoablation is used in the treatment of residual tumors after resection.

A key aspect is the complete removal of the lesion while maintaining an adequate margin of healthy tissue, ensuring oncological cleanliness of the surgical site. PMTs are not encapsulated and are locally invasive, requiring a sufficiently wide excision margin. Although surgical resection is considered effective in over 80% of cases (5), recurrence may occur (1). There are also known cases of recurrences in distant sites (18) (19).

## 5 Conclusion

For the practicing clinician, TIO remains a diagnostic challenge, requiring a high index of suspicion and broad clinical thinking. This case highlights that persistent musculoskeletal symptoms and laboratory abnormalities may go unexplained for extended periods despite multiple evaluations, emphasizing the crucial role of clinicians in coordinating care, considering rare conditions and directing appropriate diagnostic pathways.

A small, slowly growing tissue mass may conceal a systemically active and potentially dangerous tumor. A key issue is recognizing the relationship between local changes and biochemical disturbances, particularly hypophosphatemia.

Complete surgical removal remains the only curative therapy, leading to complete recovery and normalization of phosphate metabolism. Effective tumor localization using a combination of ^68^Ga-DOTA-TATE PET/CT and MRI underscores the importance of multimodal imaging in identifying these often difficult-to-diagnose lesions. Early recognition through interdisciplinary collaboration among surgeons, endocrinologists, histopathologists, and radiologists is essential for prompt diagnosis and treatment. This teamwork among specialists is crucial to ensuring optimal patient care and effective treatment, ultimately improving quality of life.

Long-term follow-up should focus on monitoring serum phosphate, alkaline phosphatase and FGF23 levels to ensure sustained biochemical remission and detect any recurrence early. This case highlights the importance of early diagnosis to avoid years of misdiagnosis. This is particularly significant for patients who struggle with debilitating symptoms due to a rare but entirely curable condition.

## Data Availability

The original contributions presented in the study are included in the article/supplementary material. Further inquiries can be directed to the corresponding author.
